# Clinical implications of different risk factor profiles in patients with mesenteric venous thrombosis and systemic venous thromboembolism: a population-based study

**DOI:** 10.1007/s11239-019-01816-x

**Published:** 2019-02-12

**Authors:** Saman Salim, Moncef Zarrouk, Johan Elf, Anders Gottsäter, Signy Sveinsdottir, Peter Svensson, Stefan Acosta

**Affiliations:** 1 Department of Clinical Sciences, Malmö, Lund University, Malmö, Sweden; 2 Department of Translational Medicine, Lund University, Malmö, Sweden; 3 Vascular Centre, Department of Cardio-Thoracic and Vascular Surgery, Skåne University Hospital, 205 02 Malmö, Sweden; 4 Centre of Thrombosis and Haemostasis, Skåne University Hospital, Malmö, Sweden

**Keywords:** Mesenteric venous thrombosis, Venous thromboembolism, Thrombophilia testing, Factor V Leiden mutation, Prothrombin mutation

## Abstract

It is unknown whether the risk factor profile for mesenteric venous thrombosis (MVT) is different from systemic venous thromboembolism (VTE). The aim of the present population-based study was to compare acquired and inherited risk factors in MVT versus VTE. Identification of all MVT patients at Skåne University Hospital between 2000 and 2015 was performed in patient records and *AuriculA* (Swedish anticoagulation registry). VTE patients were retrieved from the Malmö Thrombophilia Study (MATS), including 1465 consecutive unselected VTE patients between 1998 and 2008. Patients with MVT (n = 120) were younger (p < 0.001), had higher glomerular filtration rate (p < 0.001), lower smoking rate (p < 0.001), and had less often undergone recent surgery (p = 0.025). The prevalence of solid cancer (19.2% in MVT versus 12.1% in VTE; p = 0.026) and intra-abdominal cancer (16.7% versus 2.3%; p < 0.001) were higher in MVT. The prevalence of factor V Leiden mutation without presence of cancer was lower in MVT compared to VTE (26.6% versus 38.9%; p = 0.031). Thirty-day mortality was higher in the MVT group (9.2% versus 0.6%; p < 0.001), but did not differ at long-term follow-up according to Kaplan–Meier analysis (p = 0.73). Patients with MVT have a higher prevalence of cancer and lower prevalence of factor V Leiden mutation than those with systemic VTE. Intra-abdominal cancer should be excluded in MVT patients, and the high prevalence of factor V Leiden mutation in patients without cancer in both groups suggests that screening for thrombophilia in patients without cancer should be considered in this population for both groups.

## Highlights

- Risk factor profiles in MVT and VTE has never been compared in the same population.
- MVT patients had a higher prevalence of solid and intra-abdominal cancer.
- VTE patients had higher prevalence of factor V Leiden mutation.
- Factor V Leiden mutation prevalence in patients without cancer was high in both groups suggesting that screening for thrombophilia should be considered.
- The study findings should be externally validated in another population.

## Introduction

Mesenteric venous thrombosis (MVT) is a rare and potentially lethal disease [1]. Unspecific abdominal pain is often present in the early stage of the disease, whereas localized abdominal pain develops later. Melena, hematemesis, or hematochezia occurs in only 15%, whereas occult bleeding may be present in 50% of the cases [2]. However, there is rarely any clinical suspicion of MVT, and diagnosis may come as a surprise for clinicians after radiological imaging [3]. Main causes of MVT are coagulation disorders, abdominal inflammatory conditions, malignancies, and liver diseases [4]. When to perform thrombophilia testing in patients with MVT, and how to interpret the results, is debatable. Importantly, since there is a considerable morbidity and mortality associated with MVT, concern and anxiety regarding the underlying cause may lead to testing for thrombophilia in many patients. MVT may also be the first clinical manifestation of myeloproliferative neoplasms [5]. Although inherited and acquired thrombophilias are acknowledged to increase the risk of systemic venous thromboembolism (VTE) some authors argue that the majority of patients with systemic VTE should not be tested for thrombophilia [6]. It is unknown whether the risk factor profile for MVT is the same as for systemic venous thromboembolism (VTE). However, since population based studies on both MVT [7] and systemic VTE [8] have been performed in Malmö, Sweden, there was a unique opportunity to analyse differences in risk factor profile between these two venous thrombotic groups. The aim of the present population-based study was to compare acquired and inherited risk factors in MVT versus VTE, assuming that the risk factor profile would be similar in both groups.

## Methods

### Retrieval of patients with mesenteric venous thrombosis

Identification of all MVT patients treated surgically or conservatively at Skåne University Hospital between 1st of January 2000 and 31st of December 2015 was performed in patients records and *AuriculA* (Swedish quality registry for patients treated with anticoagulation; [9]), and based on the International Statistical Classification of Diseases and Related Health Problems (ICD), tenth edition, codes I81 (portal vein thrombosis [PVT] or MVT) and K55 (mesenteric ischemia). All patient records as well as unclear cases of mesenteric ischemia were scrutinized and validated. Only patients with symptomatic thrombosis in the superior mesenteric vein with or without anatomical involvement of portal or splenic vein, diagnosed by radiological imaging (computed tomography [CT]), laparotomy and/or autopsy, were included in the present study. Patients with liver disease were included. Myeloproliferative disease and other malignancies were present or diagnosed at the time of MVT diagnosis. Full thrombophilia panel with eight tests including Janus kinase 2 v617F mutation (JAK2) [7] was available for 74% in the MVT cohort. End of follow-up for MVT patients was September 6, 2017. Median and mean follow up time were 5.4 and 6.2 years, respectively, and interquartile range [IQR] was 2.0–10.6 years.

### Retrieval of patients with venous thromboembolism

The Malmö Thrombophilia Study (MATS) is a prospective population-based study conducted at Skåne University Hospital in Malmö, a city of 300.000 inhabitants in southern Sweden. This is the only hospital in the area treating patients with venous thromboembolism (VTE). The MATS cohort includes 1465 consecutive unselected VTE patients that were followed after inclusion in this study (March 1998) until death or the end of the study (September 2017) [10]. Thirteen patients with portal and/or mesenteric vein thrombosis were excluded from this cohort, but those with CT verified MVT were included in the MVT cohort. Seventy percent of all patients treated for VTE at Skåne University Hospital were included in the study. The remaining 30% were excluded due to unwillingness to participate, language barrier, dementia or other severe illness that prevented the patient from participating. The patients had to have objectively verified deep venous thrombosis (DVT) and/or pulmonary embolism (PE) with phlebography, duplex ultrasound, computed tomography (CT), lung scintigraphy or magnetic resonance imaging (MRI). Other inclusion criteria in MATS were age > 18 years and ability to communicate in the Swedish language. All participants provided written informed consent and the study were approved by the Lund University Ethical Committee (Dnr 2015/143). All patients were treated in accordance to the standard treatment protocol of Skåne University Hospital. Included patients were required to submit blood samples, answer a questionnaire and were evaluated concerning risk factors for VTE. Malignancies were present or diagnosed at the time of VTE diagnosis. No documentation of myeloproliferative disease was done. End of follow-up for VTE patients was September 6, 2017. Median and mean follow up time were 11.4 and 10.2 years, respectively, and IQR was 6.5–13.7 years.

The DNA mutations for factor V Leiden and Prothrombin were analysed using Taqman allele discrimination with gene specific assays for the two factors (Applied Biosystems, Life Technologies Corporation, Carlsbad, CA, USA).

### Definitions

Glomerular filtration rate (GFR) was calculated as a simplified variant of Modification of Diet in Renal Disease Study Group (MDRD).

### Statistics

Data management and statistical analysis were performed using the SPSS for Windows programme package (SPSS version 22.0, Chicago, IL, USA). Distribution of variables was expressed with median value and IQR. Differences in proportions were evaluated using the Chi square or the Fisher’s exact test. Quantitative differences between groups were assessed with the Mann–Whitney U test. Cumulative survival was analysed using the Kaplan–Meier method and life table analysis. Log rank test was used in the overall comparison of survival curves for the MVT versus systemic VTE group. Patients were censored for death in both groups until end of follow-up, September 6, 2017. A p-value < 0.05 was considered significant.

## Results

### Comparison of patient characteristics and acquired risk factors in patients with mesenteric venous thrombosis versus systemic VTE

Patients with MVT (n = 120; all symptomatic) were younger (p < 0.001), had higher glomerular filtration rate (93 ml/min versus 67 ml/min; p < 0.001), lower prevalence of smoking (p < 0.001), and had less often undergone recent surgery (p = 0.025) compared to patients with systemic VTE. In six individuals with median age 75 years (IQR 60–82) fatal MVT was detected at autopsy. Previous VTE tended to be more prevalent in patients with MVT (p = 0.072). The prevalences of cancer (19.2% in MVT versus 12.1% in VTE; p = 0.026) and intra-abdominal cancer (16.7% in MVT versus 2.3% in VTE; p < 0.001) were both higher in MVT (Table 1). Of nine patients with myeloproliferative neoplasm in the MVT group, eight (89%) were JAK-2 mutation positive. The prevalences of cast therapy, trauma and immobilization in the VTE cohort were 3.9% (57/1452), 8.2% (119/1452) and 17.1% (248/1452), respectively.

**Table 1:** Comparison of patient characteristics and acquired risk factors in patients with mesenteric venous thrombosis versus systemic VTE

Variable	MVT	Systemic VTE	p value
Number of patients	120	1452	
Median age (IQR); years	58 (47–70)	66 (53–76)	< 0.001
Female sex (%)	53 (44.2)	739 (50.9)	0.16
GFR (ml/min)	93 (74–136) (n = 114)	67 (52–79) (n = 970)	< 0.001
Platelet count (× 10^9^/L)	260 (177–340) (n = 112)	244 (204–299) (n = 1411)	0.35
Ongoing VTE prophylaxis (%)	2/116 (1.7)	30 (2.1)	0.80
*Acquired risk factors (%)*	82/107 (76.6)	1186/1396 (85.0)	0.022
Previous venous thromboembolism (any)	24/120 (20.0)	203/1451 (14.0)	0.072
BMI ≥ 30 kg/m^2^	24/88 (27.3)	296/1364 (21.7)	0.22
Smoking (ex or current)	36/103 (35.0)	771/1346 (57.3)	< 0.001
Surgical intervention (≤ 6 weeks)	8/117 (6.8)	207 (14.3)	0.025
Long travel (≥ 3 h)	7/117 (6.0)	102 (7.0)	0.67
Malignancy (solid cancer)	23 (19.2)	176 (12.1)	0.026
Intra-abdominal malignancy	20 (16.7)	33 (2.3)	< 0.001
Hormone therapy (female only)	7/53 (13.2)	161/739 (21.8)	0.14
Pregnancy	0/53 (0)	17/739 (2.3)	0.62
None of these acquired risk factors	25/107 (23.4)	210/1396 (15.0)	0.022
Strong provocative risk factor (recent surgery or malignancy)	28/119 (23.5)	356 (24.5)	0.81

### Comparison of inherited thrombophilia in tested patients with mesenteric venous thrombosis versus systemic VTE

The prevalence of factor V Leiden mutation was lower in patients with MVT compared to patients with systemic VTE (24.7% versus 37.6%; p = 0.015). The prevalence of factor V Leiden mutation without presence of cancer was also lower in MVT compared to VTE (26.6% versus 38.9%; p = 0.031). There was no difference in prevalence of the prothrombin (PT) mutation between the two groups (Table 2).

**Table 2:** Comparison of inherited thrombophilia in tested patients with mesenteric venous thrombosis versus systemic VTE

Variable	MVT	Systemic VTE	p value
Number of patients	120	1452	
Heterozygous FVL mutation (%)	19/89 (21.3)	348/1021 (34.1)	0.014
Homozygous FVL mutation (%)	3/89 (3.4)	36/1021 (3.5)	0.94
FVL mutation (any) (%)	22/89 (24.7)	384/1021 (37.6)	0.015
FVL mutation (any) without malignancy (%)	21/79 (26.6)	360/926 (38.9)	0.031
Heterozygous PT mutation (%)	3/89 (3.4)	58/1259 (4.6)	0.79
Homozygous PT mutation (%)	0/89 (0.0)	0/1259 (0.0)	–
PT mutation (any) (%)	3/89 (3.4)	58/1259 (4.6)	0.79
Compound FVL and PT mutation (%)	0/89 (0.0)	11/1245 (0.9)	1.0
FVL or PT mutation (any) (%)	25/89 (28.1)	429/1036 (41.4)	0.014
No FVL or PT mutation (%)	64/89 (72.0)	605/1036 (58.4)	0.013

*FVL* Factor V Leiden, *PT* prothrombin

### Comparison of survival in patients with mesenteric venous thrombosis versus systemic VTE

**Fig. 1:**
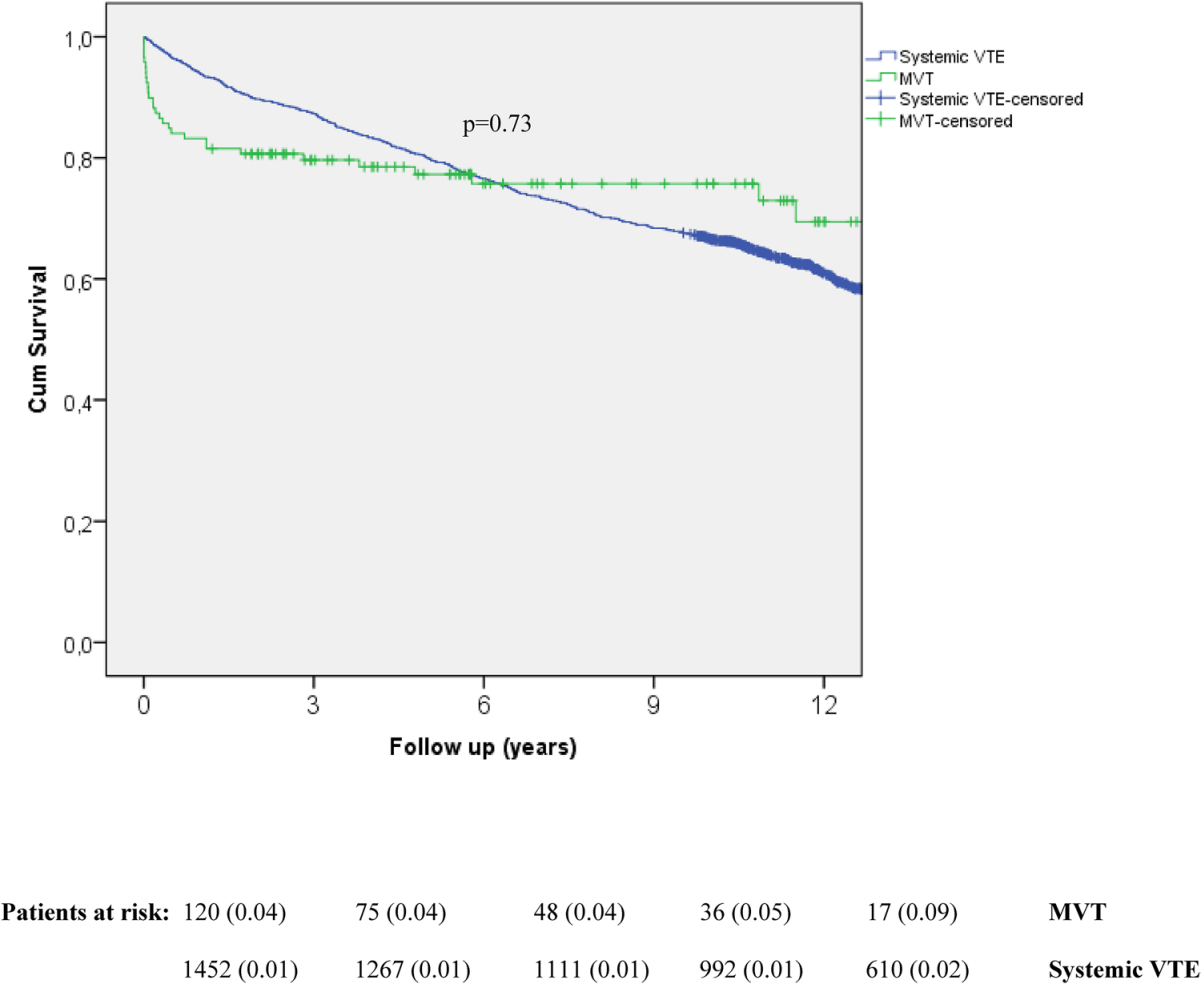
Kaplan–Meier analysis of long-term survival in patients with mesenteric venous thrombosis (MVT) and systemic venous thromboembolism (VTE). Life table showing patients at risk at each time point. Standard error of cumulative proportion surviving at end of interval is stated within parentheses. Censored patients are marked with ticks

Thirty-day mortality was higher in the MVT group (10.8% versus 0.5% in VTE; p < 0.001), but did not differ at long-term follow-up according to the Kaplan–Meier analysis (p = 0.73) (Fig. 1). The cause of the 13 deaths in the MVT group at 30 days were the following: Intestinal ischaemia (n = 9), liver cirrhosis (n = 2), pulmonary embolism (n = 2) and pancreatic cancer with metastasis (n = 2). Among these 13 deaths, eight patients underwent clinical autopsy and additional two underwent bowel resection during surgery. The cause of the seven deaths in the VTE group at 30 days were the following: Metastatic cancer (pulmonary [2], pancreatic [1] and unknown [1]) disease (n = 4), operation for gastric cancer (n = 1), pulmonary embolism (n = 3), acute myocardial infarction with multiple arterial embolization (n = 2) and cerebral haemorrhage (n = 1). Among these seven deaths, three underwent clinical autopsy and additional one was operated upon. As multiple causes of death were registered for some patients, the number of causes exceeds the number of patients in each group.

## Discussion

Patients with MVT and systemic VTE have different risk factor profile as shown in this population-based comparative study. Patients with MVT have a higher prevalence of cancer, and the present study data suggests that intra-abdominal cancer should simultaneously be excluded at the diagnostic CT examination of the abdomen for MVT. Screening for occult cancer in the chest, breast, cervix or prostate, showed a low prevalence of occult cancer in patients with first unprovoked systemic VTE, not increasing after adding a CT examination of the abdomen and pelvis [11]. The high prevalence of factor V Leiden mutation without presence of cancer in both groups, 27% in MVT and 39% in systemic VTE, suggests that screening for thrombophilia may be considered in both study groups. The much higher 30-day mortality of 10.8% in the MVT group, mostly caused by intestinal infarction, is of particular concern, and anxiety of recurrence of MVT, development towards intestinal infarction and death, may lead to both unselected screening for thrombophilia and consideration of life-long anticoagulation treatment, especially in the absence of a sole reversible risk factor such as first episode of acute pancreatitis and trauma [12, 13].

The vast majority of patients will receive indefinite anticoagulation treatment due to their high MVT related mortality [1, 4]. For patients in whom the decision of indefinite anticoagulation is made due to the presence of a non-reversible strong risk factor, such as active cancer, further thrombophilia testing has no clinical consequences. The principle of indefinite treatment in patients without detection of any risk factor is in line with current American College of Chest Physicians guidelines for VTE, recommending indefinite anticoagulation treatment for patients “with a first VTE that is an unprovoked proximal DVT of the leg or PE and who have a low or moderate bleeding risk” [14].

Importantly, testing for thrombophilia including both inherited factors such as Factor V Leiden mutation, prothrombin gene mutation, and deficiencies of protein C, protein S, antithrombin, and acquired thrombophilic factors such as Janus kinase 2 v617F (JAK2) mutation, lupus anticoagulant, and cardiolipin antibodies is not expensive in relation to other diagnostic tests. Current price at the present study centre is 302 € [15]. The duration of anticoagulation therapy in patients with an identified non-reversible provoking factor such as Factor V Leiden mutation is a matter of debate on the other hand [4]. In a population-based study including 900 VTE patients, patients with heterozygous FVL mutation had an increased risk (Odds ratio 2.4) for new VTE recurrence during a mean follow up of 5 years [8]. The high rate of inherited and acquired prothrombotic factors present in patients with MVT [7] and potential severe clinical consequences of recurrence makes experts tend to offer patients with identified laboratory-confirmed thrombophilia indefinite anticoagulation, despite low level of evidence. Consequently, routine laboratory screening may be considered in patients with MVT without an identified provocative factor on CT scan. The European Society of Vascular Surgery guidelines recommend lifelong anticoagulation in patients with MVT with proven thrombophilia [4].

The limitations of the study are attributed to the retrospective design of data collection in patients with MVT, whereas systemic VTE data in MATS were prospectively registered. The finding that MVT patients were less likely smokers than patients with systemic VTE might have been attributed to younger age [16] and retrospective data sampling in the MVT group. The younger age of MVT patients is more difficult to explain taking into account that the prevalence of cancer, which increases with age [17], was higher in this group, and that an aged subgroup of six individuals with MVT detected at autopsy were included in the MVT group. This age discrepancy between the two groups needs to be externally validated in another comparative cohort study. In fact, it seems likely that younger age in the MVT group is a factor contributing to the absence of mortality difference at long-term.

Only Factor V Leiden and prothrombin mutation was documented for the systemic VTE patients in MATS, whereas a full thrombophilia panel with eight tests including JAK2 mutation [7] was available for 74% in the MVT cohort. In contrast to the prevalence of heterozygous FVL mutation, the small sample size of patients with homozygous FVL mutation makes evaluation of differences in prevalence between the two groups impossible. It would have been very interesting to evaluate differences in prevalence of JAK2 mutation and clinical consequences between these two groups, considering the relative high incidence of myeloproliferative neoplasm in the MVT group [5]. A prospective large nationwide cohort study with sufficient number of patients with both MVT and systemic VTE is needed to evaluate differences in risk factor profiles of other thrombophilias than factor V Leiden and prothrombin mutation. Novel candidate markers for venous thrombosis such as plasminogen activator inhibitor-1 should then be considered in the test panel [18, 19]. Since thrombophilia profiles may vary greatly in different populations [7, 20], the fact that the compared cohorts are from the same population constitutes an important strength of the present study.

In conclusion, patients with MVT have different risk factor profile than those with systemic VTE; higher prevalence of cancer and lower prevalence of factor V Leiden mutation. Intra-abdominal cancer should be excluded in MVT patients, and the high prevalence of factor V Leiden mutation without presence of cancer in both groups suggests that screening for thrombophilia in patients without cancer should be considered in this population for both groups unless the clinician beforehand irrespective of thrombophilia can decide to give indefinite anticoagulation.
